# Effects of PM_2.5_ on Skeletal Muscle Mass and Body Fat Mass of the Elderly in Taipei, Taiwan

**DOI:** 10.1038/s41598-019-47576-9

**Published:** 2019-08-01

**Authors:** Chi-Hsien Chen, Li-Ying Huang, Kang-Yun Lee, Chih-Da Wu, Hung-Che Chiang, Bing-Yu Chen, Wei-Shan Chin, Shih-Chun Pan, Yue Leon Guo

**Affiliations:** 1 Department of Environmental and Occupational Medicine, National Taiwan University Hospital Hsin-Chu Branch, Hsinchu, Taiwan; 2 Department of Environmental and Occupational Medicine, National Taiwan University (NTU) College of medicine and NTU Hospital, Taipei, Taiwan; 3 School of Medicine, College of Medicine, Fu Jen Catholic University, New Taipei City, Taiwan; 4 Division of Endocrinology and Metabolism, Department of Internal Medicine, and Department of Medical Education, Fu Jen Catholic University Hospital, Fu Jen Catholic University, New Taipei City, Taiwan; 5 Department of Internal Medicine, School of Medicine, College of Medicine, Taipei Medical University, Taipei, Taiwan; 6 Division of Pulmonary Medicine, Department of Internal Medicine, Shuang Ho Hospital, Taipei Medical University, New Taipei City, Taiwan; 7 Department of Geomatics, National Cheng Kung University, Tainan, Taiwan; 8 National Institute of Environmental Health Sciences, National Health Research Institutes, Miaoli County, Taiwan; 9 Department of Medical Research and Development, Chang Gung Memorial Hospital, Keelung, Taiwan; 10 School of Nursing, College of Nursing, Taipei Medical University, Taipei, Taiwan; 11 Institute of Occupational Medicine and Industrial Hygiene, National Taiwan University, Taipei, Taiwan

**Keywords:** Risk factors, Epidemiology, Skeletal muscle

## Abstract

Loss of skeletal muscle mass is common with aging and can cause morbidity and mortality in the elderly. The effects of particulate air pollution on skeletal muscle mass is not known. The study aims to assess the chronic effects of ambient fine particulates (PM_2.5_) on the body composition of the elderly. From October 2015 to November 2016, a cross-sectional survey on 530 elderly (age > = 65 years) was conducted in the Taipei Basin, Taiwan. The body composition was measured by bioelectrical impedance analysis (InBody 120). One year exposure to air pollution was estimated using the Kriging method at the participant’s residence. Multiple linear regression analysis, after adjustments for demographics and co-pollutants, was used to examine the effects of PM_2.5_ on body composition indices and force of handgrip. Changes in body composition for an interquartile (1.4 μm/m^3^) increase in PM_2.5_ concentration included a 0.4 kg (95% confidence interval (CI): −0.31, −0.58; p < 0.0001) decrease in skeletal muscle mass (2.0%) and a 0.7 kg (95% CI: 0.47, 0.91; p < 0.0001) increase in body fat mass (3.6%). While PM_2.5_ reduced fat free mass in the upper extremities and trunk, but not in the lower extremities, it increased body fat mass in the three parts. There was no significant effect of PM_2.5_ on handgrip force. Higher physical activity (versus lower than median) was associated with less detrimental effect of PM_2.5_ on skeletal muscle mass and body fat mass (p values for interaction term: 0.009 and 0.013, respectively). Long-term PM_2.5_ exposure is associated with decreased skeletal muscle mass and increased body fat mass in the elderly, which can be ameliorated by physical activity.

## Introduction

Loss of muscle mass is associated with aging, at a decreasing rate of approximately 6% per decade after mid-life^1^. Aging is associated with decreased production of several anabolic hormones like growth hormone, insulin-like growth hormone, testosterone, and estrogen, all of which play important roles in maintaining muscle mass and strength^2^. Secondary causes of muscle loss include inadequate nutrition, physical inactivity, and chronic diseases such as malignancy, organ dysfunction, and neurodegenerative or endocrine diseases^3^. Extremely low muscle mass with inadequate muscle function in the elderly is referred to as sarcopenia, which is linked to mortality risk^4,5^. When sarcopenia is accompanied by increased body fat mass, also known as sarcopenic obesity, mortality risk becomes higher comparing to each condition separately^6–8^. Identifying and managing the risk factors for sarcopenia and sarcopenic obesity may help promote healthy aging.

Although the precise cause of sarcopenia and sarcopenic obesity is not known, there are several patho-physiologic mechanisms that affect muscle loss and visceral fat gain. Insulin resistance, a common phenomenon of central obesity, may induce muscle fiber atrophy, intra-muscular lipid accumulation, and mitochondrial dysfunction. These enhance muscle wasting, dysfunction and oxidative stress^2,3,9^. Sarcopenia further exacerbates obesity-associated insulin resistance and dysglycemia^10^. Moreover, local or systemic inflammatory states driven by pro-inflammatory cytokines or oxidative stress can enhance proteolysis and inhibit muscle synthesis^11–13^. The over-deposition of adipose tissue, especially in visceral site, augments pro-inflammatory cytokines (i.e. TNFα and IL-6) and have negative effect on muscles^14^. Loss of muscle mass and functionality may reduce physical activity, which in turn lowers energy expenditure and boosts the development of obesity^3^. Such mechanisms may set a vicious cycle between sarcopenia and obesity.

Particulate air pollution, especially particulate matter smaller than 2.5 μm (PM_2.5_), are known to augment systemic inflammation, insulin resistance, and oxidative stress. In a mice study, PM_2.5_ increases inflammation in adipose tissue and decreases glucose uptake in muscular tissue, resulting in increased systemic insulin resistance^15^. However, there is paucity of information regarding the effect of ambient PM_2.5_ on human skeletal muscle and adipose tissue. Previous studies show that cigarette smoke leads to skeletal muscle cell damage, muscle protein breakdown^16^, skeletal muscle dysfunction^17^, and central obesity^18^. Because ambient PM_2.5_ and cigarette smoke share some common patho-physiologic mechanisms like oxidative stress and inflammation, ambient PM_2.5_ may have negative effects on muscle and adipose tissue in the elderly, a population susceptible to air pollution.

## Materials and Methods

### Study design and population

Between October 2015 and November 2016, a cross-sectional study on the elderly (age > = 65 years) was conducted in the Taipei Basin, Taiwan. Those who underwent their annual health exam in two hospitals were invited. Those with malignancy or ambulation and communication difficulties were excluded. The institutional review board of the National Health Research Institutes (EC1040508-E-R2) approved this study. All of the study participants provided a written informed consent. All experiments were performed in accordance with relevant guidelines and regulations.

### Questionnaire

A structured questionnaire was designed to collect information on personal habits (e.g. cigarette smoking and alcohol drinking), medical conditions (e.g. underling diseases and corresponding treatments), education, and physical activity. The Chinese edition of the Physical Activity Scale for the Elderly (PASE) questionnaire was used to assess physical activity^19,20^. Two well-trained interviewers provided the questionnaire. Because of visual impairment and reading difficulties among the elderly, the interviewers helped in reading and explaining the questions and in filling up the answers.

### Measurements of body composition and handgrip force

All of the participants underwent measurements of body composition in the morning after >8 hours of fasting to avoid the influence of food and fluid intake^21^. The participants were in a standing posture and lightly dressed for the examination. Bioelectric impedance analysis devices (BIA, Inbody 120, InBody Co., Ltd. Seoul, Korea) with tetrapolar-8-point-tactile electrodes were used. Each device had two different frequencies (20 and 100 kHz) for impedance measurement in five body segments (the four extremities and the trunk).

The body composition parameters used were skeletal muscle mass (SMM; in kg) of total body, fat free mass (FFM; in kg) of five body segments, and body fat mass (BFM; in kg) of the total body and of the five body segments. The FFM and BFM of the upper or lower extremities were calculated by adding up the component weights of the right and left sides of the arms or legs. Height and weight were measured at the time of body composition measurement.

Handgrip strength was measured using a digital handgrip-dynamometer (TTM-YD, Tokyo, Japan). Three trials for each hand were performed and the best reading was used for data analysis.

### Assessment of air pollution exposure

The residential address of each participant was geocoded to estimate the ambient air pollution exposure. The annual mean concentrations of air pollutants, including PM_2.5_, sulphur dioxide (SO_2_), ozone (O_3_), carbon monoxide (CO), and nitrogen dioxide (NO_2_), in 73 EPA monitoring stations in Taiwan in 2015 were calculated based on hourly measured data. A modified ordinary Kriging adopted from Liao *et al*.^22^ was used to approximate the long-term residential exposure. ArcView GIS (version 93) and its Geostatistical Analysts Extension (ESRI Inc., Redland, CA) were used to construct the semi-variogram for spatial estimation of the concentrations of the air pollutants. The cross-validated R^2^ values of PM_2.5_, NO_2_, CO, O_3_, and SO_2_ were 0.61, 0.63, 0.28, 0.20, and 0.61, respectively.

### Statistical analysis

Linear regression for association between individual variables, muscle strength, and parameters of body composition, and Pearson’s correlation for the relationship between each air pollutant were calculated using the JMP software version 5.0 (SAS Institute, Gary, NC, USA).

Multiple linear regression analysis was used to examine the association between air pollution and health outcomes. The models were adjusted for covariates such as age (years), sex (male/female), body height (cm), body weight (kg), cigarette smoking (never/former/current), alcohol drinking habit (none/less than once per week/more than once per week), education (primary school or less/middle or high school or equivalent/university degree or more), physical activity (total score on the PASE questionnaire), diabetes mellitus (yes/no), asthma (yes/no), COPD (yes/no), stroke history (yes/no), heart diseases (yes/no), chronic renal disease (yes/no), arthritis (yes/no), osteoporosis (yes/no), and ambient temperature and humidity on the date of examination. Personal estimates of long-term exposure to air pollutant at residential the sites were fitted separately into the multiple linear regression model. The two-pollutant model was also applied to adjust for potential confounding effects of co-pollutants. Stratified analysis were done to examine potential modification effect of age (<or> = median), gender, physical activity (PASE score <or> = median), physician diagnosed diabetes mellitus (yes or no), physician diagnosed arthritis (yes or no), and physician diagnosed osteoporosis (yes or no). Interaction term in the full model was used to evaluate the statistical significance of modification effects. A *p* < 0.05 was considered statistically significant.

## Results

### The characteristics of participated elders

**Figure 1:**
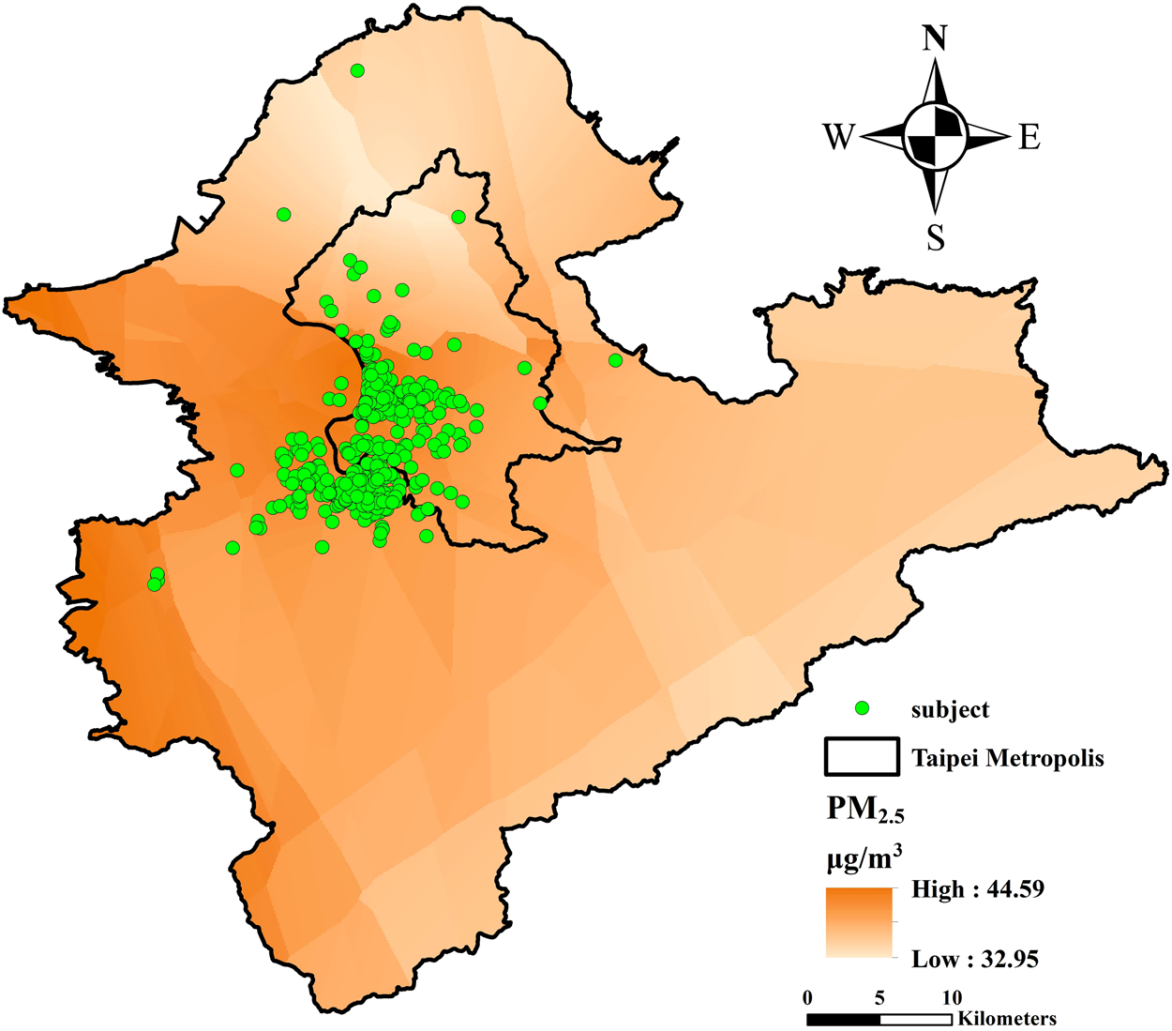
The residential locations of the 530 study subjects and the Kriging estimation of ambient PM_2.5_ in 2015.

The study population had a mean age of 70.2 years, with male-female ratio of 0.71. Their mean BMI was 24.09 kg/m^2^ and 36.6% had a BMI > 25 kg/m^2^. Among them, 5.1% were current smokers and 9.8% were frequent alcohol drinkers (more than once per week). The prevalence of chronic diseases was highest for arthritis (13.8%), followed by diabetes mellitus (12.8%) and osteoporosis (9.1%). The total score of PASE ranged from 0 to 286.2, with mean and median values of 107.4 and 103, respectively. The demographics, body composition indices, and handgrip force of the participants were summarized in Table 1, while their residential locations were shown in Fig. 1.

**Table 1:** Characteristics of the elderly/participants of the study (n = 530).

	Mean (SD) or %
Age, yr	70.21 (3.92)
Male sex, %	41.51
Body height, cm	158.21 (7.80)
Body weight, kg	60.48 (10.12)
Body mass index	24.09 (3.10)
Education level, %	
Low (primary school or less)	25.47
Medium (middle or high school or equivalent)	56.60
High (university degree or more)	17.93
Smoking status, %	
Never	83.77
Current	5.09
Former	11.13
Alcohol drinking	
No alcohol drinking habit	65.28
<1 per week	24.91
≥1 per week	9.81
Physician diagnosed diseases	
Asthma, %	1.51
COPD, %	2.45
Diabetes mellitus, %	12.83
Stroke, %	3.40
Heart diseases, %	7.36
Renal diseases, %	2.26
Arthritis, %	13.77
Osteoporosis, %	9.06
PASE scores	107.38 (50.81)
Hand grip force, kg	25.39 (8.69)
Body composition indices	
Skeletal muscle mass, kg	22.38 (4.60)
Body fat mass, kg	19.26 (5.70)
Fat free mass of upper extremities, kg	4.13 (1.19)
Fat free mass of trunk, kg	18.28 (3.67)
Fat free mass of lower extremities, kg	12.40 (2.86)
Body fat mass of upper extremities, kg	2.66 (1.03)
Body fat mass of trunk, kg	9.62 (3.08)
Body fat mass of lower extremities, kg	5.90 (1.62)

Abbreviations: PASE, physical activity scale for the elderly.

### The distribution of air pollution at residential sites

The distributions of estimated concentrations of five air pollutants among the participants were shown in Table 2. The mean PM_2.5_ at residential locations was 18.1 μg/m^3^, which exceeded the National Ambient Air Quality Standards of Taiwan (15 μg/m^3^). The mean concentrations of NO_2_, CO, O_3_, and SO_2_, were below the national regulated levels. Moreover, NO_2_ was highly correlated with CO (r = 0.89) but negatively correlated with O_3_ (r = −0.73) (Table 3).

**Table 2:** Distributions of air pollution exposure at the residences of the study participants (n = 530).

	Mean	Median	IQR	Minimum	Maximum
Exposures in 2015					
PM_2.5_, μg/m^3^	18.08	17.93	1.41	12.23	22.37
NO_2_, ppb	21.11	21.29	0.32	16.08	21.62
CO, ppm	0.72	0.72	0.01	0.55	0.73
O_3_, ppb	26.3	26.11	0.55	25.8	30.4
SO_2_, ppb	3.22	3.22	0.05	2.87	3.47

Abbreviations: IQR, inter-quartile range; PM_2.5_, particulate matter with aerodynamic diameter of 2.5 μm; NO_2_, nitrogen dioxide; CO, carbon monoxide; O_3_, ozone; SO_2_, sulphur dioxide.

**Table 3:** Correlations of air pollutants in 2015 at the residences of the study participants (n = 530).

	PM_2.5_	NO_2_	CO	O_3_	SO_2_
PM_2.5_	1	0.179	0.272	−0.253	0.306
NO_2_		1	0.887	−0.73	0.473
CO			1	−0.58	0.151
O_3_				1	−0.385
SO_2_					1

Abbreviations: IQR, inter-quartile range; PM_2.5_, particulate matter with aerodynamic diameter of 2.5 μm; NO_2_, nitrogen dioxide; CO, carbon monoxide; O_3_, ozone; SO_2_, sulphur dioxide.

### The effect of air pollution on body composition

The association between air pollution exposure and body composition was shown in Table 4. The 2015 average of PM_2.5_ at the residential locations was associated with lower skeletal muscle mass and higher body fat mass. An interquartile increase of 1.41 μg/m^3^ of PM_2.5_ reduced skeletal muscle mass by 0.4 kg (95% confidence interval (CI): −0.31, −0.58; p < 0.0001), and increased fat mass by 0.7 kg (95% CI: 0.47, 0.91; p < 0.0001), representing 2.0% and 3.6% of change respectively. In terms of the effect of air pollutant on various body parts, PM_2.5_ reduced the skeletal muscle mass of the upper extremities and trunk by 5.1% and 3.1%, respectively, but not the lower extremities. On the other hand, PM_2.5_ increased body fat mass in each part (5.7% for the upper extremities, 2.2% for the trunk, and 5.6% for the lower extremities).

**Table 4:** Estimated correlation coefficients of body composition indices and handgrip force for each ambient air pollutant.

	Grip force	SMM	BFM
PM_2.5_	−0.087 (0.270)	−**0**.**442** (**0**.**069**)^**a**^	**0**.**690** (**0**.**113**)^**a**^
NO_2_	0.069 (0.129)	0.002 (0.035)	−0.0008 (0.056)
CO	0.089 (0.132)	−**0**.**076** (**0**.**035**)^**d**^	**0**.**121** (**0**.**057**)^**d**^
O_3_	−0.071 (0.216)	0.006 (0.058)	−0.013 (0.094)
SO_2_	0.018 (0.185)	0.057 (0.049)	−0.083 (0.080)
	**FFMU**	**FFMT**	**FFML**
PM_2.5_	**−0**.**212** (**0**.**022**)^**a**^	**−0**.**569** (**0**.**059**)^**a**^	−0.055 (0.039)
NO_2_	0.006 (0.012)	0.017 (0.031)	0.002 (0.019)
CO	**−0**.**034** (**0**.**012**)^**c**^	**−0**.**089** (**0**.**031**)^**c**^	−0.007 (0.019)
O_3_	0.005 (0.019)	0.003 (0.051)	0.005 (0.032)
SO_2_	0.033 (0.017)	0.089 (0.044)	0.035 (0.027)
	**BFMU**	**BFMT**	**BFML**
PM_2.5_	**0**.**151** (**0**.**025**)^**a**^	**0**.**215** (**0**.**059**)^**b**^	0.329 (0.039)^a^
NO_2_	0.002 (0.012)	0.004 (0.028)	−0.005 (0.020)
CO	**0**.**029** (**0**.**012**)^**d**^	0.040 (0.029)	**0**.**056** (**0**.**020**)^**c**^
O_3_	−0.005 (0.020)	−0.010 (0.048)	−0.0004 (0.033)
SO_2_	−0.023 (0.017)	−0.025 (0.041)	−0.039 (0.028)

^a^*p* < 0.0001; ^b^*p* < 0.001; ^c^*p* < 0.01; ^d^*p* < 0.05.

Models were adjusted for age, sex, body height, body weight, smoking, alcohol drinking, educational attainment, physical activity, physician-diagnosed diseases (i.e., diabetes, asthma, COPD, stroke, heart, renal, arthritis, osteoporosis), temperature, and humidity.

Regression coefficients and standard errors were estimated for every inter-quartile range increase in each pollutant.

Abbreviations: SMM, skeletal muscle mass; BFM, body fat mass; FFMU, fat free mass of upper extremities; FFMT, fat free mass of trunk; FFML, fat free mass of lower extremities; BFMU, body fat mass of upper extremities; BFMT, body fat mass of trunk; BFML, body fat mass of lower extremities; PM_2.5_, particulate matter with aerodynamic diameter of 2.5 μm; NO_2_, nitrogen dioxide; CO, carbon monoxide; O_3_, ozone; SO_2_, sulphur dioxide.

### Two-pollutant model to clarify the most hazardous air pollutant

In the single pollutant model (Table 4), there were mild effects of CO on skeletal muscle mass and body fat mass, with a similar pattern to the effects of PM_2.5_. The two-pollutant model was therefore adjusted for the confounding effect of co-pollutants (Table 5). The effect of PM_2.5_ on body composition was consistent even after adjustments for the co-pollutants. However, the effect of CO became statistically non-significant after adjustments for PM_2.5_ (Table 5).

**Table 5:** Association between air pollutants and body composition indices using the two-pollutant model.

	SMM	BFM	FFMU	FFMT	BFAU	BFMT	BFML
PM_2.5_							
with NO_2_	**−0**.**460**^**a**^	**0**.**716**^**a**^	**−0**.**223**^**a**^	**−0**.**597**^**a**^	**0**.**156**^**a**^	**0**.**221**^**b**^	**0**.**343**^**a**^
with CO	**−0**.**430**^**a**^	**0**.**670**^**a**^	**−0**.**208**^**a**^	**−0**.**558**^**a**^	**0**.**145**^**a**^	**0**.**207**^**b**^	**0**.**320**^**a**^
with O_3_	**−0**.**477**^**a**^	**0**.**742**^**a**^	**−0**.**228**^**a**^	**−0**.**614**^**a**^	**0**.**161**^**a**^	**0**.**229**^**b**^	**0**.**356**^**a**^
with SO_2_	**−0**.**532**^**a**^	**0**.**826**^**a**^	**−0**.**258**^**a**^	**−0**.**692**^**a**^	**0**.**183**^**a**^	**0**.**256**^**a**^	**0**.**393**^**a**^
CO							
with PM_2.5_	−0.023	0.039	−0.009	−0.020	0.011	—	0.017
with NO_2_	**−0**.**378**^**a**^	**0**.**595**^**a**^	**−0**.**190**^**a**^	**−0**.**507**^**a**^	**0**.**134**^**a**^	—	**0**.**297**^**a**^
with O_3_	**−0**.**113**^**c**^	**0**.**178**^**d**^	**−0**.**049**^**b**^	**−0**.**133**^**b**^	**0**.**042**^**c**^	—	**0**.**085**^**b**^
with SO_2_	**−0**.**085**^**d**^	**0**.**133**^**d**^	**−0**.**038**^**c**^	**−0**.**101**^**c**^	**0**.**032**^**d**^	—	**0**.**062**^**c**^

^a^*p* < 0.0001; ^b^*p* < 0.001; ^c^*p* < 0.01; ^d^*p* < 005.

The models were adjusted for age, sex, body height, body weight, smoking, alcohol drinking, educational attainment, physical activity, physician-diagnosed diseases (e.g., diabetes, asthma, COPD, stroke, heart, renal, arthritis, osteoporosis), temperature, and humidity.

Regression coefficients and standard errors were estimated for every inter-quartile range increase in each pollutant.

Abbreviations: SMM, skeletal muscle mass; BFM, body fat mass; FFMU, fat free mass of upper extremities; FFMT, fat free mass of trunk; FFML, fat free mass of lower extremities; BFMU, body fat mass of upper extremities; BFMT, body fat mass of trunk; BFML, body fat mass of lower extremities; PM_2.5_, particulate matter with aerodynamic diameter of 2.5 μm; NO_2_, nitrogen dioxide; CO, carbon monoxide; O_3_, ozone; SO_2_, sulphur dioxide.

Of the 530 relatively healthy elderly who participated in this study, there was no statistically significant effect of ambient air pollution on handgrip force (Table 4).

### The modification effect of physical activity

In stratified analysis, we found physical activity significantly modified the PM_2.5_ effect on body compositions, the lower the physical activity the more the loss of skeletal muscle mass and the increase in body fat mass related to PM_2.5_ exposure (Table 6).

**Table 6:** Associations between PM_2.5_ exposures, skeletal muscle mass and body fat mass, stratified by personal factors.

	Skeletal muscle mass, kg				Body fat mass, kg			
	coeff.	se	p	p interaction	coeff.	se	p	p interaction
Age								
<median	−0.564	0.108	<0.0001	0.095	0.893	0.175	<0.0001	0.079
> = median	−0.345	0.094	0.0003		0.520	0.152	0.0007	
Sex								
female	−0.393	0.086	<0.0001	0.285	0.623	0.139	<0.0001	0.383
male	−0.505	0.119	<0.0001		0.762	0.193	0.0001	
PASE								
<median	−0.601	0.102	<0.0001	0.009	0.937	0.168	<0.0001	0.013
> = median	−0.313	0.096	0.001		0.495	0.155	0.002	
DM								
Yes	−0.517	0.207	0.016	0.354	0.799	0.336	0.022	0.372
No	−0.411	0.076	<0.0001		0.644	0.123	<0.0001	
Arthritis								
Yes	−0.537	0.253	0.038	0.760	0.856	0.406	0.039	0.717
No	−0.443	0.073	<0.0001		0.690	0.120	<0.0001	
Osteoporosis								
Yes	−0.511	0.223	0.029	0.533	0.784	0.361	0.038	0.518
No	−0.449	0.074	<0.0001		0.703	0.120	<0.0001	

Abbreviations: PASE, physical activity scale for the elderly.

## Discussion

This study demonstrates, for the first time, that exposure to ambient PM_2.5_ is associated with a reduction in skeletal muscle mass and an increase in body fat mass in the Taiwanese elderly, a population vulnerable to the effects of air pollution and to sarcopenia. Such effects are consistent even after adjusting for co-pollutants. Long-term (average of one year) PM_2.5_ exposure reduces muscle mass mainly on the upper extremities and trunk, but not for the lower extremities. On the other hand, ambient PM_2.5_ is also associated with increased body fat mass in the same body parts. Physical activity ameliorates the detrimental effect of PM2.5 on body composition.

In this study, ambient PM_2.5_ has a detrimental effect on muscle mass, particularly on the non-weight bearing muscles. Compared to previous research, where the average decrease in muscle mass is 6% per decade after mid-life^1^, the dose effect of PM_2.5_ in this study is approximately a 2.0% reduction in skeletal muscle mass for every 1.4 μg/m^3^ increase in PM_2.5_. This is a noteworthy issue. Although the mechanisms involved in muscle wasting among the elderly are probably multi-factorial and still poorly understood, chronic inflammation and insulin resistance are the probable explanations^2^. Many experimental and epidemiologic evidences have demonstrated the effects of ambient PM_2.5_ on exacerbating systemic inflammation and insulin resistance^23^, providing the biological explanation for the observed effects in this study.

In addition, the non-observed effects of PM_2.5_ on the lower extremities, the weight-bearing body part, and the protective role of physical activity, imply that exercise may ameliorate the deleterious effects of PM_2.5_ on muscle mass. Because all of the study participants have full ambulation function, general weight bearing and walking may provide the basic resistance and aerobic training activities for their lower limbs. A previous study applying a short course exercise program for elderly with sarcopenia shows that both resistance and aerobic training can increase muscle mass and strength^24^, suggesting that exercise may be an effective way to overcome the pathologic process of sarcopenia. Further study with a longitudinal design and exercise intervention on non-weight bearing limbs may help to clarify how body activity modifies the detrimental effect of PM_2.5_.

There were also significant effects of PM_2.5_ on increasing body fat mass. Previous animal studies have shown the direct effects of ambient PM_2.5_ exposure on adipose tissue^15,25,26^. In mammals, adipocytes are classified into two types: white adipose tissue, or the primary site of energy storage, and brown adipose tissue, which are specialized for fatty acid metabolism, energy expenditure, and heat generation^27^. In mice studies, long-term exposure to PM_2.5_ impairs the function of brown adipose tissue and changes the gene expression from brown to white adipocyte specific patterns^25,26^. Previous literature has linked the reduced functionality of brown adipose tissue to the propensity for obesity^28^. Such aforementioned evidences may explain how PM_2.5_ influences the development of obesity. Because fat tissue expansion can further increase insulin resistance and pro-inflammatory states^3^, leading to more muscle wasting^2,11–14^, the differential or possibly even synergetic effects of PM_2.5_ on muscle and fat tissue established in this study may enhance the pathologic process toward sarcopenia.

Although there was no significant effect of PM_2.5_ on handgrip force, it may be too premature to conclude that ambient PM_2.5_ has no effect on muscular function. This study enrolled the elderly mostly living in the Taipei Basin, where the range of long-term PM_2.5_ exposure is not wide. This may hamper the detectability of PM_2.5_-associated changes in muscular functionality. Future studies enrolling participants living in areas with higher and wider ranges of air pollution exposure may provide a better picture for examining the dose-response relationship.

There are several limitations to our study. First of all, our study has chosen the Kriging interpolation rather than the satellite-based approach^29–31^ or land-use regression^32^ for exposure assessments. This may lead to certain degree of non-differential misclassification bias. Yet due to the meteorological conditions and cloud contamination in Northern Taiwan, the satellite-based aerosol optical depth measurements have high missing rates in Taipei. This hampers the utilization of the satellite-based approach in our study. Furthermore, the published land-use regression model^33^ for PM_2.5_ in Taiwan was based on data from automatic beta attenuation measurements. However, this method can be interfered by the high humidity in Taiwan^34^. Accordingly, the Taiwan EPA has decided to report PM_2.5_ concentration by manual sampling using the US Federal Reference Method as our standard methods since 2014. Moreover, the urbanization similarity and monitoring station density are high in Taipei, the misclassification bias by Kriging method should be small. (There are 16 monitoring stations in the Taipei Basin with approximately area of 243 km^2^. Most of our study subjects resided in a 15 km * 15 km area with 13 monitoring stations.) Owing to aforementioned reasons, our study finally decided to use the Kriging interpolation for PM_2.5_ concentration estimation. This method may not totally avoid misclassification bias, but the influence may be trivial and acceptable. Also, a significant exposure response trend between quartile of PM_2.5_ exposure and body composition parameters is found, which supports the existence of the correlation (see Supplementary Fig. S1).

Secondly, since our study has used a cross-sectional design, precaution has to be taken in determining a causal relationship. Further longitudinal studies are needed to confirm the effect of PM_2.5_ on declining muscle mass by age. To minimize the potential confounding effect on causal inference, this study statistically controlled several co-variables and chronic disease status related to body composition in previous literature. In particular, physical activity has been adjusted by applying the total score of the Physical Activity Scale for the Elderly, a value indicating daily activity-related caloric expenditure.

Lastly, the limitation of not controlling the nutrition intake should be noted. Because only generally healthy elderly were enrolled, or those who could walk to the hospital on their own to get the regular health exam, it is also likely that they can get their daily food without difficulty. Financial problems in buying daily food are also less likely in this study population because Taiwanese elderly have monthly paid pension, which fulfills their basic daily requirement. Also, there are different effects of PM_2.5_ on muscle and on fat tissues, and on the upper and lower limb muscles. These are less likely to be explained by nutritional status since malnutrition reduces both muscle and fat mass throughout the entire body.

In conclusion, this study reveals that long-term exposure to urban PM_2.5_ is associated with reduced skeletal muscle mass and increased body fat mass among healthy elderly living in Taipei Basin, where the annual average of PM_2.5_ concentration is much higher than the regulated levels suggested by the World Health Organization and where traffic emission is the main source of ambient fine particles. Physical activity ameliorates the detrimental effect of PM_2.5_ on skeletal muscle mass and body fat mass. Further studies with a longitudinal design and conducted in areas with higher and wider ranges of PM_2.5_ exposure are warranted to verify the causal relationship and determine the effects on muscle function.

## Supplementary information

Supplementary figure
