# Supplementary material for: Effects of PM2.5 on Skeletal Muscle Mass and Body Fat Mass of the Elderly in Taipei, Taiwan
Source: Sci Rep. 2019 Aug 1;9:11176. doi: 10.1038/s41598-019-47576-9 (PMC6671961; doi:10.1038/s41598-019-47576-9)
Supplement: Supplementary file 1 — Supplementary figure [file 41598_2019_47576_MOESM1_ESM.docx]

**Supplementary Information.**

**Title:**

**Effects of PM_2.5_ on Skeletal Muscle Mass and Body Fat Mass of the Elderly** **in Taipei, Taiwan**

**Authors:**

Chi-Hsien Chen^1,2^, Li-Ying Huang^3,4^, Kang-Yun Lee^5,6^, Chih-Da Wu^7^, Hung-Che Chiang^8^, Bing-Yu Chen^9^, Wei-Shan Chin^10^, Shih-Chun Pan^11^, and Yue Leon Guo^2,8,11^

**Authors’ details**

^1^Department of Environmental and Occupational Medicine, National Taiwan University Hospital Hsin-Chu Branch, Hsinchu, Taiwan.

^2^Department of Environmental and Occupational Medicine, National Taiwan University (NTU) College of medicine and NTU Hospital, Taipei, Taiwan

^3^School of Medicine, College of Medicine, Fu Jen Catholic University, New Taipei City, Taiwan

^4^Division of Endocrinology and Metabolism, Department of Internal Medicine, and Department of Medical Education, Fu Jen Catholic University Hospital, Fu Jen Catholic University, New Taipei City, Taiwan

^5^Department of Internal Medicine, School of Medicine, College of Medicine, Taipei Medical University, Taipei, Taiwan

^6^Division of Pulmonary Medicine, Department of Internal Medicine, Shuang Ho Hospital, Taipei Medical University, New Taipei City, Taiwan

^7^Department of Geomatics, National Cheng Kung University, Tainan, Taiwan

^8^National Institute of Environmental Health Sciences, National Health Research Institutes

^9^Department of Medical Research and Development, Chang Gung Memorial Hospital, Keelung, Taiwan

^10^School of Nursing, College of Nursing, Taipei Medical University, Taipei, Taiwan

^11^Institute of Occupational Medicine and Industrial Hygiene, National Taiwan University, Taipei, Taiwan

**Corresponding Author:**

Prof. Yue Leon Guo

Department of Environmental and Occupational Medicine
College of Medicine

National Taiwan University and

National Taiwan University Hospital

Rm 339, 17 Syujhou Road, Taipei 100, Taiwan

Phone: +886-2-3322-8216

Fax: +886-2-3322-8214

E-mail: [leonguo@ntu.edu.tw](mailto:leonguo@ntu.edu.tw)

**Supplementary Figure Legends**

**Supplement figure S1.** Relationship between quartile of fine particulate matter exposure and body composition parameters.

p<0.0001 by trend test

p<0.0001 by trend test
